# Supplementary figures and images for: Survivorship and feeding preferences among size classes of outplanted sea urchins, Tripneustes gratilla, and possible use as biocontrol for invasive alien algae
Source: PeerJ. 2015 Sep 15;3:e1235. doi: 10.7717/peerj.1235 (PMC4579015; doi:10.7717/peerj.1235)

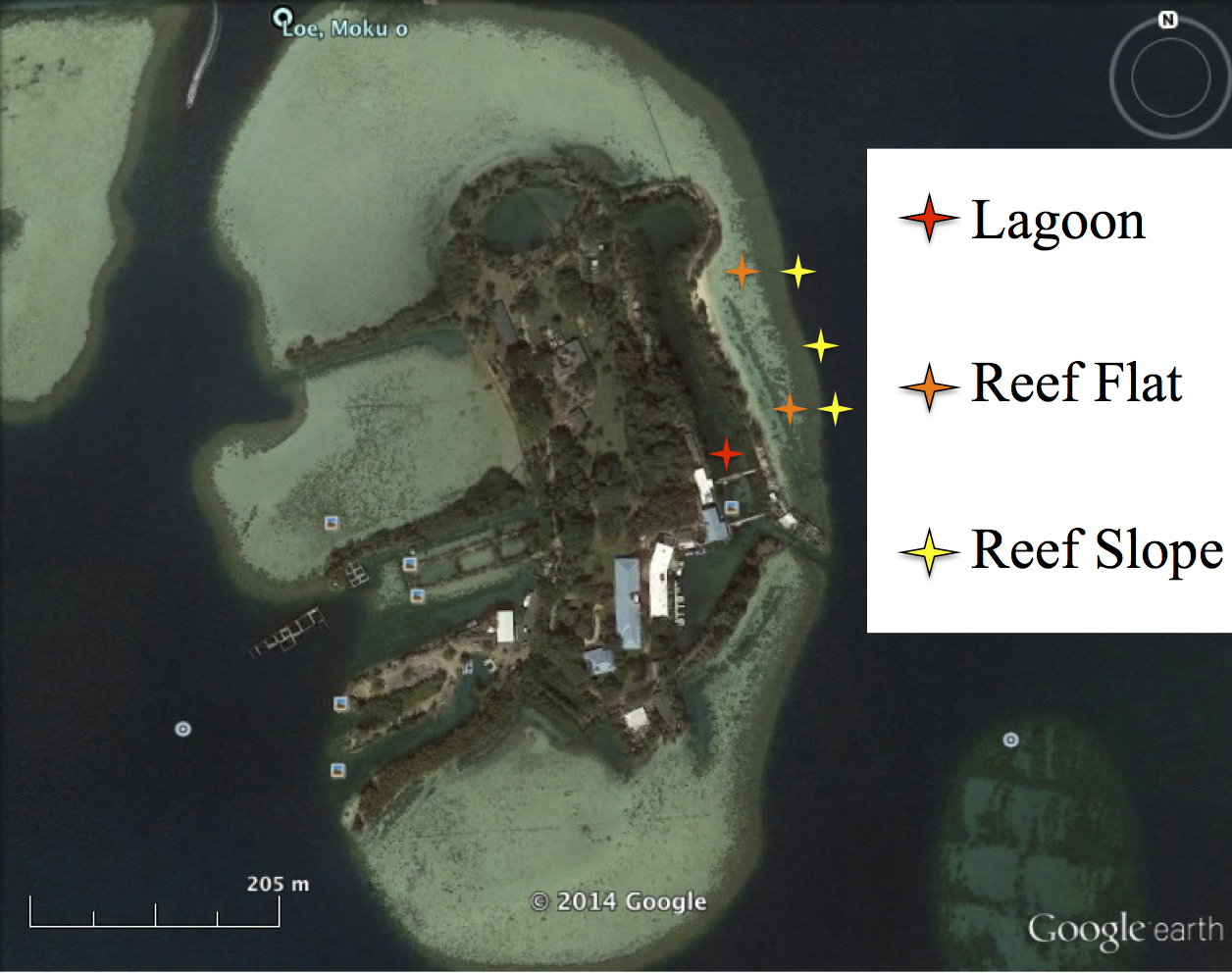

Supplement: Supplemental Information 1 — Location of caging experiment around Coconut Island in Kāneʻohe Bay. The red star corresponds to our lagoonal site (low water motion). The orange stars represent are caging sites along the reef flat (high water mixing, low predation). And the yellow stars mark caging areas along the reef slope (mixing, higher predation). These three sites were used to assess post transport survival of juvenile urchins which are deployed in the Bay. [file peerj-03-1235-s001.png]

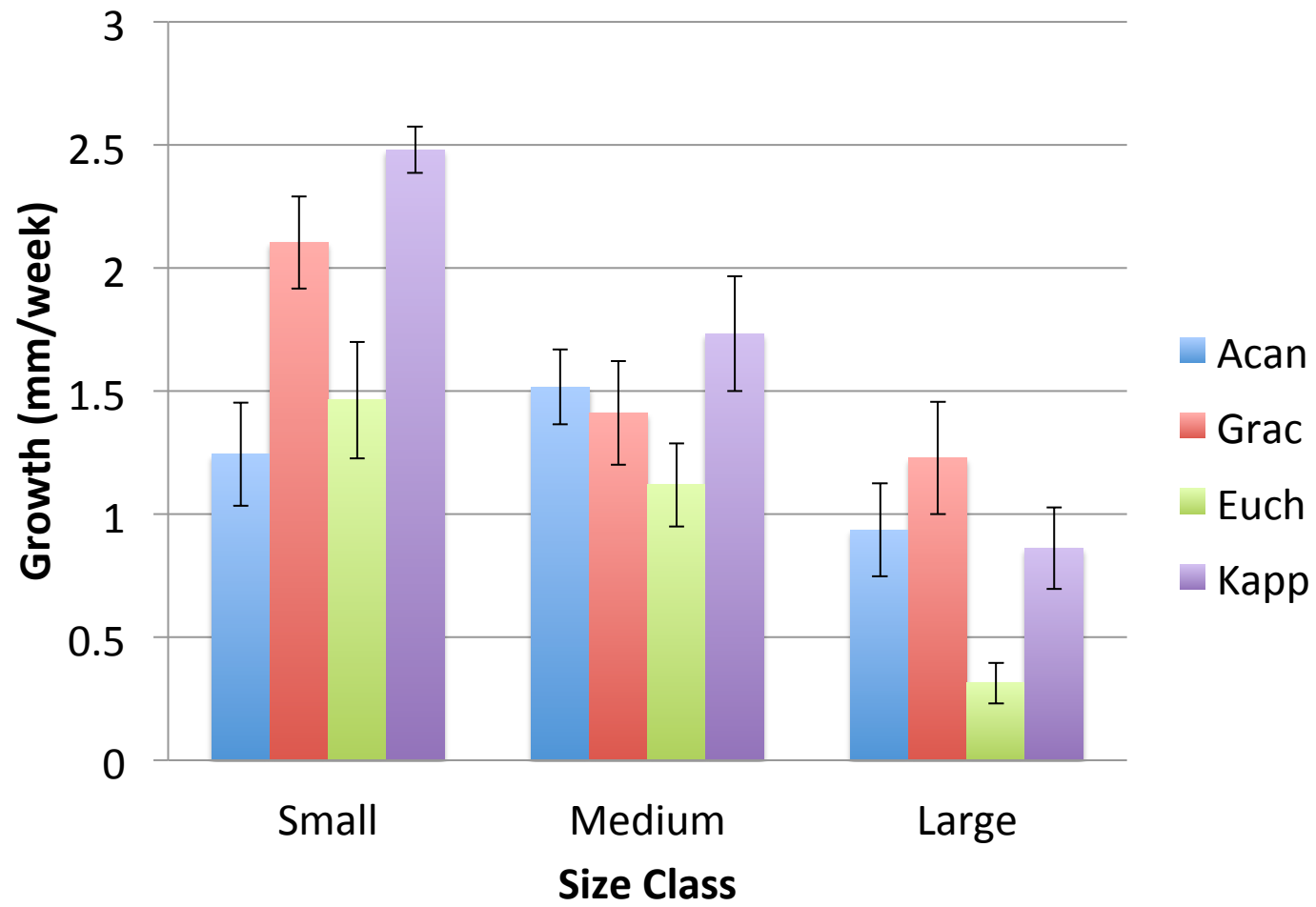

Supplement: Supplemental Information 2 — Average growth rates of small, medium and large urchins while on a no-choice diet of either A. spicifera, G. salicornia, E. denticulatum or K. clade B. [file peerj-03-1235-s002.pdf]
